# Development of Mucoadhesive Chitosan Derivatives for Use as Submucosal Injections

**DOI:** 10.3390/polym10040410

**Published:** 2018-04-06

**Authors:** Hidemi Hattori, Masayuki Ishihara

**Affiliations:** 1 Department of Biochemistry and Applied Biosciences, Faculty of Agriculture, University of Miyazaki, 1-1 Gakuenkibanadai-nishi, Miyazaki 889-2192, Japan; h-hattori@cc.miyazaki-u.ac.jp; 2 Division of Biomedical Engineering, Research Institute, National Defense Medical College, 3-2 Namiki, Tokorozawa, Saitama 359-8513, Japan

**Keywords:** chitosan derivatives, mucoadhesive, mucopolysaccharide, endoscopic submucosal dissection, endoscopic mucosal resection, submucosal injection

## Abstract

Endoscopic mucosal resection (EMR) and endoscopic submucosal dissection (ESD) have been used for surgical treatment of early gastric cancer. These endoscopic techniques require proper submucosal injections beneath the tumor to provide a sufficiently high submucosal fluid cushion (SFC) to facilitate clean dissection and resection of the tumor. Until now, the submucosal injection materials developed for endoscopic techniques such as EMR and ESD of tumors have been composed of macromolecules, proteins, or polysaccharides. We have been investigating the use of chitosan, a product that is obtained by the alkaline deacetylation of chitin, the second-most abundant natural polysaccharide. Specifically, we have been studying a photocrosslinked chitosan hydrogel (PCH) and solubilized chitosan derivatives for use as novel submucosal injections for endoscopic techniques. Notably, chitosan derivatives with lactose moieties linked to the amino groups of its glucosamine units can specifically interact with acidic mucopolysaccharides and mucins in submucosa without the need for the incorporation of harmful photoreactive groups nor potentially mutagenic ultraviolet irradiation.

## 1. Introduction

Gastric cancers are typically classified into early and advanced stages. Early-stage cancers are defined as those in which lesions have infiltrated up to the submucosa without metastasis into the lymph node; in contrast, advanced-stage cancers exhibit submucosal infiltration and metastasis. For patients with early-stage lesions, endoscopic surgeries are expected to increasingly be applied to reduce medical costs and physical burdens [1,2,3].

Several techniques have been developed for the endoscopic mucosal resection (EMR) of large superficial lesions of the gastrointestinal (GI) tract, using various natural, synthetic, and semisynthetic materials such as chitosan and their derivatives [4,5]. Although saline-assisted EMR has been established as a minimally invasive therapy, *en bloc* resection and histopathological analyses are required to monitor for complete removal of the tumor in question. In addition, complete resection of lesions of more than 2 cm in diameter remains difficult, despite improved EMR techniques [6,7]. Application of endoscopic submucosal dissection (ESD) has improved dissection rates for *en bloc* resection of large lesions, but excessive bleeding and perforation occurs more frequently during ESD than during EMR [3,7]. 

The success of these endoscopic techniques relies on proper submucosal injections beneath the tumor, providing a submucosal fluid cushion (SFC) sufficient to facilitate clean dissection and aresection of the tumor. Various types of submucosal injection agents have been developed and employed in EMR and ESD, including normal physiological saline, hypertonic saline, dextrose, glycerol, and others; each agent has distinct advantages and disadvantages [4,8]. 

Chitin, a natural polymer of *N*-acetylglucosamine, is the second-most abundant polysaccharide in nature (after cellulose) and is obtained primarily from crustacean or shrimp exoskeletons. Chitosan, comprising *N*-acetylglucosamine and glucosamine, can be obtained by alkaline deacetylation of chitin and has been found to be nontoxic and biocompatible with living tissue [9,10,11]. Chitosan is positively charged owing to the generation of free amino groups. The degree of deacetylation and molecular weight of the product affect the solubility, hydrophobicity, viscosity, and electrostatic properties of chitosan, with the electrostatic properties affecting the polymer’s ability to interact with polyanions through the protonated amino groups [11,12]. Chitosan and its derivatives have attracted considerable interest due to their biological activities, which include antimicrobial, antitumor, and hypocholesterolemic functions, along with stimulation of wound healing [13,14]. Chitosan is commonly used in biomedical materials for wound dressings, artificial skin, hemostasis, and adhesion, and as a raw material for cosmetic and antibacterial agents. However, chitosan is only soluble in acidic solvents such as diluted hydrochloric acid, acetic acid, propionic acid, and ascorbic acid, making this reagent difficult to handle as a biomaterial in wound dressings and biological adhesion treatments [14]. Numerous studies have attempted to improve the water solubility of chitosan over a broad pH range to provide the polymer with advanced functionalities [15,16]; modifications have included changes to carbohydrate branching [17] and derivatization using different types of disaccharides, including lactose, maltose, and cellobiose [18]. Furthermore, chemically modified chitosans have been investigated for improved solubility, with modifications including *O*-carboxymethylation [19], *O*-hydroxyethylation [20], *N*-hydroxypropylation [21], and *N*-succinylation [22]. 

We prepared a photocrosslinkable chitosan derivative (Az-CH-LA) [23,24] containing both 2% lactose moieties (lactobionic acid) and 2.5% photoreactive azide groups (*p*-azidobenzoic acid) on the free amino groups. Az-CH-LA exhibited high aqueous solubility at neutral pH. Az-CH-LA generated insoluble photocrosslinked chitosan hydrogel (PCH) after cross-linking induced by ultraviolet (UV) irradiation. In other contexts, PCH has been used as a drug delivery material for topical cancer treatments [25,26]. However, the hydrogelation of PCH remains a hindrance to its wider application, requiring a photoreactive azide group (*p*-azidobenzoic acid) as crosslinker and the use of UV irradiation, which potentially has harmful effects on normal tissues. Therefore, further modification of our chitosan (to permit use without the crosslinker and UV irradiation) would be required before this reagent could be employed safely for submucosal injection in endoscopy techniques. 

To test an example of such a possible agent, we characterized a soluble chitosan derivative containing 2% lactobionic acid on the free amino groups, demonstrating that this version exhibited good aqueous solubility, even at neutral pH [27]. We further examined this derivative’s interactions with anionic polysaccharides and proteins to determine its potential use as a submucosal injection agent for safe endoscopic techniques [27]. In the present review, we describe various submucosal injection materials, focusing on PCH and the chitosan derivatives that we have been studying.

## 2. Chitosan-Based Biomaterials as Biological Adhesives and Hemostats

Certainly, ESD is the most popular surgical treatment for early gastric cancer because it allows the resection of entire layers, including the lesion, and facilitates histopathological diagnoses. However, ESD is technically difficult and time consuming and has risks of bleeding and perforation.

Biological adhesives are used for tissue adhesion, hemostasis, and sealing of the leakage of air and body fluids during surgical procedures. Although most bleeding during surgical procedures can be controlled with suturing, hemostasis is often uncontrollable under conditions such as coagulopathy, inflammation, infection, and severe adhesion, and in the presence of anticoagulant medications [28]. In addition, intractable air leakage in lung surgery is often observed, especially in emphysematous lung disease [28]. In many cases, of uncontrollable bleeding and air leakage, hemostasis and air sealing have been addressed by using adhesives, including chemically crosslinked gelatins [29], cyanoacrylate polymers [30,31], and fibrin glues [32,33]. Such adhesives need to be locally non-irritating, systemically nontoxic, appropriately flexible, and biodegradable. However, cytotoxicity and severe tissue irritability of biological adhesives have been reported following the use of resorcinol, formaldehyde, and carbodiimides for the crosslinking of gelatins [29], or due to the formation of formaldehyde following degradation of cyanoacrylate [30,31]. Fibrin glue (Beriplast P), which contains fibrinogen, thrombin, factor XIII, and protease inhibitors, exploits blood coagulation systems to seal tissues and is currently the most widely used surgical adhesive. Accordingly, fibrin glue’s hemostatic effects have been reported by many investigators [32,33]. However, industrial production of fibrin glue requires the use of human blood. Alternatively, chitosan-based hydrogels, including curable chitosan-poly (ethylene glycol) -tyramine hydrogels [34], catechol-functionalized chitosan/pluronic hydrogels [35], and PCHs, have been proposed for tissue adhesives. 

**Figure 1:**
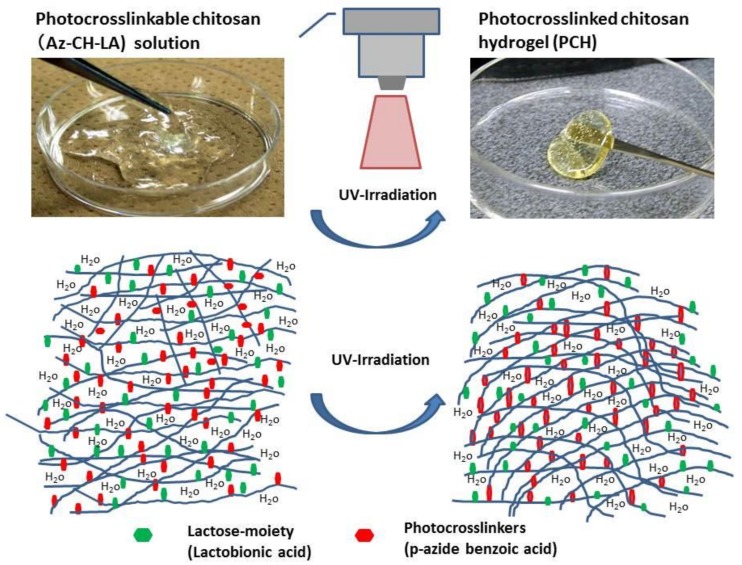
Photocrosslinkable chitosan (Az-CH-LA) solution and photocrosslinked chitosan hydrogel (PCH). The viscous Az-CH-LA solution (**upper, left photograph**) was converted to insoluble soft rubber-like PCH (**upper, right photograph**) through crosslinking reaction of the azide and amino groups of Az-CH-LA with UV-irradiation (**lower illustration**).

Chitosan, comprising *N*-acetylglucosamine and glucosamine, can be obtained by alkaline deacetylation of chitin and has been found to be nontoxic and biocompatible with living tissue [9,14]. Our laboratory has been studying a photocrosslinkable chitosan derivative, Az-CH-LA [23,24]. The chitosan used in this study had a molecular weight of 300–500 kDa with 80% deacetylation. Lactose moieties were introduced through condensation reactions of chitosan with amino groups. Chitosan containing 2% lactobionic acid (CH-LA) exhibited high aqueous solubility, even at neutral pH. Furthermore, application of ultraviolet light (UV) irradiation at a lamp distance of 2 cm (Spot Cure ML-251C/A) with a guide fiber unit (SF-101BQ) and a 250-W lamp (240–380 nm; major peak, 340 nm; Usio Electrics Co., Ltd., Tokyo, Japan) to Az-CH-LA containing 2.5% *p*-azidebenzoic acid produced an insoluble PCH within 30 s (Figure 1); this PCH could firmly adhere two pieces of ham to each other [25]. Moreover, PCH is expected to find use as a biological adhesive for tissue repair and hemostasis to seal leakages of air and body fluids during surgical procedures [23,28].

The versatility of chitosan and its derivatives offer a wide range of applications as wound dressings or scaffolds in regenerative medicine; these reagents are biodegradable, nontoxic, and can be formulated in a variety of formats, including as powders, gels, membranes, sponges, and films. Chitosan and its derivatives also can provide controlled adsorption and release of growth factors and extracellular matrix (ECM) components [36,37,38]. Additionally, given that chitosan can be readily modified to render the material compatible with a wide range of molecules, the adherent properties of chitosan can be improved to facilitate the seeding of cells. For instance, the incorporation of collagen or biologically active Arg-Gly-Asp (RGD) tripeptide-containing proteins, converting chitosan into a chitosan-collagen scaffold, has been shown to enhance the material’s cell attachment ability [39]. Furthermore, other available synthetic materials are expected to be able to be combined with chitosan to yield agents that are biocompatible with body tissues. For instance, polylactide (PLA), polyglycolide (PGA), and polylactide-*co*-glicolide (PLGA) have received much attention because of their biodegradability and biocompatibility. Conjugation of chitosan with these synthetic materials is expected to provide a potential technology for the development of desirable scaffolds for tissue regeneration [10,25,40]. 

## 3. Biomaterials for Submucosal Injection

The application of proper submucosal injections for SFCs is essential for safe ESD. A SFC lifts the mucosa to be resected, isolates lesions, and protects the muscularis propria from injury [8]. To safely perform ESD as well as EMR procedures without major complications such as perforation and excess bleeding, sufficient volumes of submucosal injection must be maintained in the submucosa until the lesion is removed. 

Various types of submucosal injection agents have been developed and employed in endoscopic surgeries such as EMRs and ESDs, each has distinct advantages and disadvantages [4,8]. Normal physiological saline, which is widely used in the clinic, is easy to inject, low in cost, and readily available; furthermore, this agent does not cause local inflammation at the sites of injection [4,5,7]. However, normal physiological saline tends to dissipate quickly during surgery. Highly viscous solutions such as hypertonic saline with epinephrine [41], hydroxypropyl methylcellulose [8], 50% dextrose [42], and glycerol are readily available, but these agents tend to cause tissue damage and local inflammation at the site of injection [4,5,7]. Other materials such as HA, chondroitin sulfate, and poloxamer 407 can help prevent such complications [43], and these materials provide the longest-lasting cushions among available agents [43,44,45]. However, HA has been shown to stimulate the growth of residual tumor cells [46]. 

Recently, several hydrogels have been described for use as alternative biomaterials, including temperature-sensitive chitosan-based injectable hydrogel (used for mimicking the cartilage matrix [47]), PEG poly (l-alanine) thermogel (used for 3D scaffolding of bone-marrow-derived mesenchymal stem cells) [48], crosslinked polydopamine/nanocellulose hydrogel (used for wound healing) [49], HA/alginate hydrogels (used for skin engineering) [50], pH-responsive tannic acid-carboxylated agarose composite hydrogel (used for wound healing) [51], catechol-functionalized chitosan/pluronic hydrogels (used as a tissue adhesive) [35], and UV crosslinked biodegradable methacrylated gelatin (used for wound healing and tissue engineering) [52]. These hydrogels have not (to our knowledge) been proposed for use as SFCs for ESD or for EMR of large superficial lesions of the GI tract. Requirements to apply as SFCs for ESD or for EMR are (1) to achieve an enough elevation at injected sites for at least a few hours, (2) to have adhesive and hemostatic activities, (3) to be biodegradable during a few months, and (4) to have stimulatory activity for wound healing as well as biocompatibility [5,7]. Furthermore, these materials are required a standard safety and toxicity tests [23,28]. However, proper materials should be evaluated and compared with each other for use in this context. One of the hydrogels described in this review might serve as an ideal injectable material for use as an SFC; such an application could facilitate the endoscopic treatment of lesions that previously could not be resected endoscopically due to size, extent, or location. 

## 4. Photocrosslinked Chitosan Hydrogel (PCH) for Submucosal Injection

**Figure 2:**
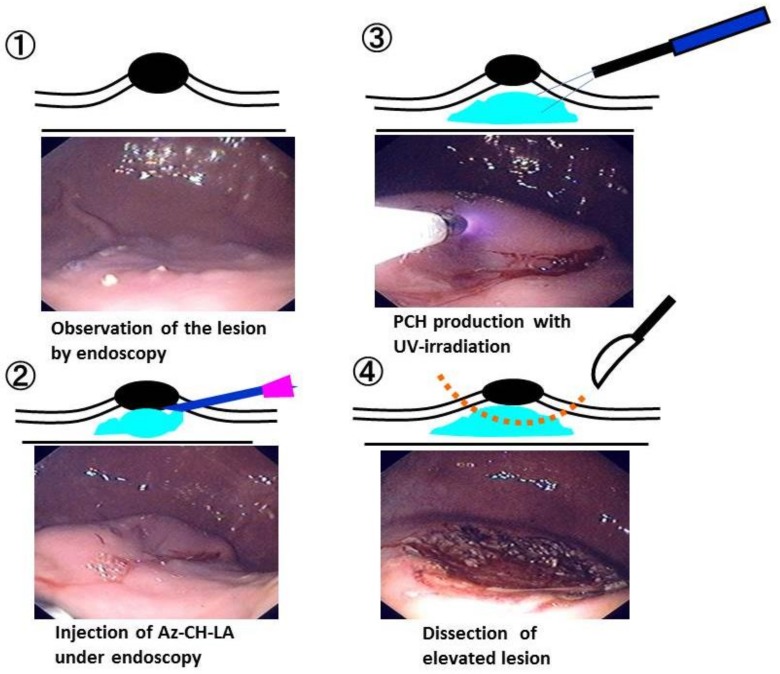
Representative images of Az-CH-LA injection, ultraviolet (UV) irradiation, and the appearance when PCH was used as SFC in PCH-assisted ESD. ① Observation of the lesion by endoscopy, ② Injection of Az-CH-LA under endoscopy, ③ PCH production with UV-irradiation, ④ Dissection of elevated lesion. These experiments demonstrated the feasibility and safety of PCH-assisted ESD in a porcine stomach model. Reprinted from [5], Copyright 2009, with permission from Thieme.

To evaluate PCH for use in submucosal injection, we performed endoscopic procedures in pigs using a single accessory channel endoscope for pigs (Olympus VQ-8143A, Tokyo, Japan) in animals that had been anesthetized and provided with general mechanical ventilation. A 23-gauge injection needle (Olympus NM-200U-0423) was passed through the endoscopic accessory channel, and mucosal elevations were created by 5-mL submucosal injections of either 10% hypertonic saline (HS; Ohtsuka Pharmaceutical Co., Tokushima, Japan) or 1% PCH. After injection of PCH, the elevated mucosa was irradiated with UV light for 30 s per 0.4 cm^2^ in a total of 10 different places, using a UV lamp system (MUV-250U-L; Moritex Co., Saitama, Japan) and UV fiber for ESD (AFP01437; Moritex Co. Japan) that also had been inserted through the endoscopic accessory channel. Submucosal injections of 1% PCH were performed through the endoscopic injection needle using a small-caliber syringe (5 mL). Figure 2 provides representative images of Az-CH-LA injections, UV irradiation, and the use of PCH as SFC in PCH-assisted ESD.

PCH injections led to sustained elevations with clearer margins than those achieved with HS and facilitated precise ESD along the margins of the elevated mucosa in the stomach [5] and esophagus [7] of pigs. The endoscopic aspect following ESD was similar in the PCH- and HS-injected groups, and biodegradation of PCH occurred within 6 weeks of the initial surgery, as demonstrated by in-life endoscopy and post-mortem histological analyses [5,7]. These experiments indicated that PCH holds promise as a SFC agent for submucosal injections prior to endoresection.

Insoluble PCH is known to prevent electrosurgical currents from entering the muscle layer. Therefore, application of PCH also is expected to prevent perforations during ESD.

**Figure 3:**
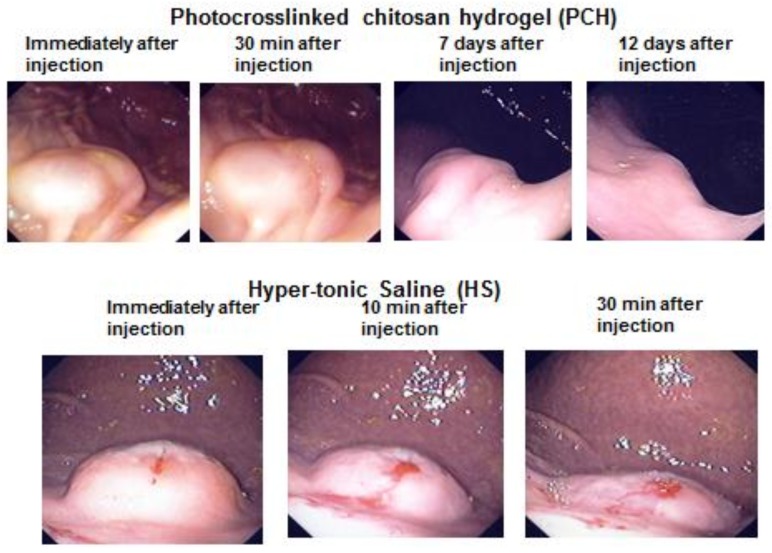
Endoscopic appearance at injection sites of PCH or HS. The change of each injection site was observed endoscopically, without dissection, at the indicated time points. Reprinted from [5], Copyright 2009, with permission from Thieme.

The PCH-assisted ESD procedure has many advantages compared with conventional ESD using HS [5,7]. The techniques for PCH-assisted ESD are simple and convenient: this method requires only injection, UV irradiation without insertion of photo-fibers, and incision without a tubular (sausage-like) light diffuser, such as that required for photodynamic therapy with Photofrin [53]. In the absence of repeated injection, PCH does not spread to the surrounding areas of the pig stomach for at least 30 min; mucosal elevations were maintained for several days following conversion to a hydrogel by exposure to UV irradiation for a total of 5 min. In contrast, elevations in HS-assisted ESD collapsed within 30 min of injection, necessitating reinjection (Figure 3). Indeed, reinjection of HS is typically required in ESD to decrease the risk of bleeding and perforation. 

**Figure 4:**
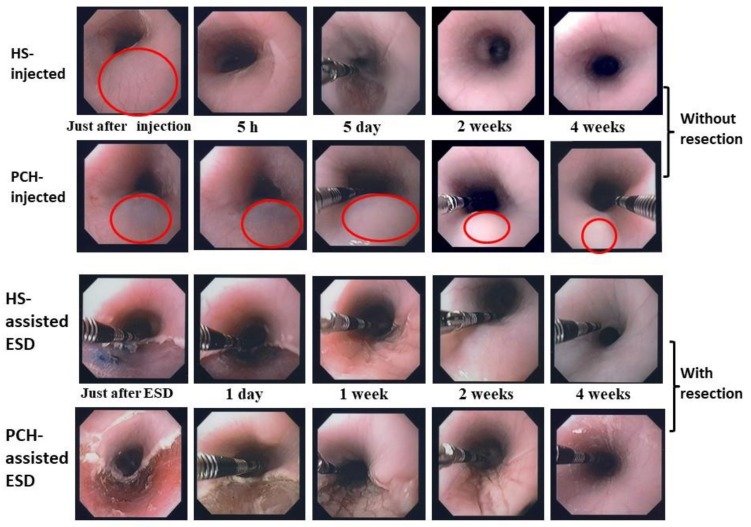
Changes in endoscopic appearance of hypertonic saline (HS)- or PCH-injected sites of pig esophagus (in live animals) without (**upper 2 rows**) and with (**lower 2 rows**) mucosal resection. Endoscopic changes of mucosal elevation shapes are shown at the following times after the injection of 2 mL HS or PCH: just after injection, and 5 h, 5 days, 2 weeks, and 4 weeks later. Endoscopic appearances are shown at the following times after HS- and PCH-assisted ESD: just after ESD, and 1 day, 1 week, 2 weeks, and 4 weeks later. Note the duration of elevation in the PCH-injected case (2nd row vs. 1st row; red circles represent centers of mucosal elevation). Reprinted from [7], Copyright 2012, with permission from Elsevier.

The PCH-assisted ESD procedure in pig esophagus was performed by endoscopic injection of 2 mL of 1% PCH and incision of the elevated mucosa (Figure 4). Control procedures were performed with HS and SH. The majority of the injected and UV-irradiated PCH could be removed along with the resected mucosa. Little residual PCH was retained in the artificial ulcers, as observed endoscopically. Moreover, after mucosal incision during PCH-assisted ESD, no bleeding or perforation was observed (by endoscopy) at sites of artificial ulcers in esophageal tissues in pigs, whereas minor bleeding and perforation was observed several times during HS- and SH-assisted ESDs [5,7]. These observations indicated that insoluble PCH may prevent electrosurgical currents from entering the muscle layer [5,7].

**Figure 5:**
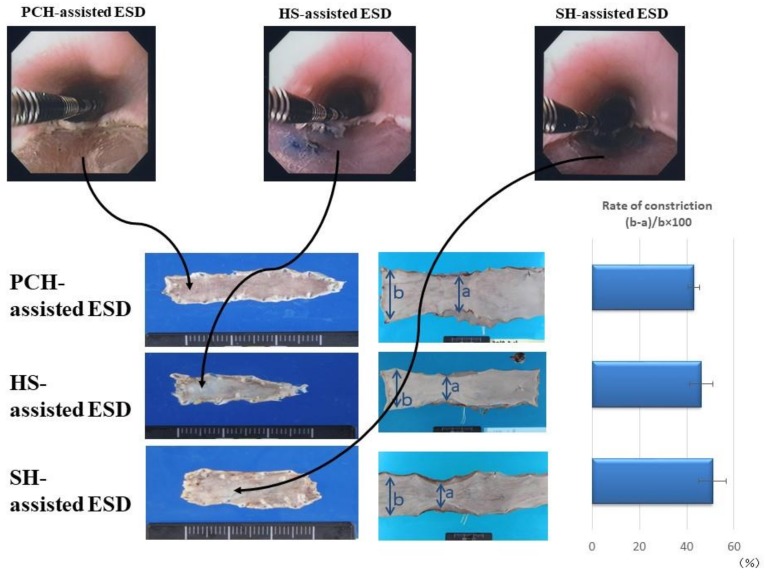
Endoscopic appearance following endoscopic submucosal dissection (ESD). (**Upper panel**)*:* Endoscopic appearance immediately after ESD assisted by photocrosslinkable chitosan hydrogel (PCH), hypertonic saline solution (HS), and sodium hyaluronate (SH) in pig esophagus in living animals (left to right, respectively). (**Lower panel**)*:* Left column: Resected tissues recovered from pig esophagi in each group, showing constriction of esophagus following ESD assisted by PCH, HS, and SH. Middle column: Resected tissues recovered from inner esophagus at 4 weeks after ESD. Right column: Calculated rate of constriction (%; mean ± SD, n = 6) at 4 weeks after ESD. Photographs are representative images from the indicated groups. Reprinted from [7], Copyright 2012, with permission from Elsevier.

The PCH-, HS-, and SH-assisted ESDs for extensive resection (more than 3 cm^2^) of the esophagus resulted in significant constriction after 2 to 4 weeks. However, PCH-assisted ESD caused nominally less constriction than did HS-assisted ESD (Figure 5). The original esophageal circumferences were approximately 50 mm at the anal side of the incised constriction region. The PCH-, HS-, and SH-assisted ESDs resulted in esophageal constriction (mean ± SD) of 42.0 ± 3.2%, 48.0 ± 6.2%, and 52.6 ± 6.7%, respectively, although these effects did not achieve statistical significance (presumably because of the small number of samples) (Figure 5).

Essentially all the injected PCH was retained in the injected submucosa for more than 4 weeks following the procedure. This interval included the formation of granulated tissue with in vivo vascularization and neutrophil infiltration. PCH in the tissue was completely degraded and replaced with tissue matrix during the subsequent 4 weeks (i.e., by 8 weeks after the procedure) [5,7]. Given that PCH shows good tissue adhesion, hemostasis, and wound repair, this hydrogel has potential as a new submucosal injectable with applications in the development of hemostasis and tissue-adherent biomaterials [54,55,56]. However, the hydrogelation of PCH remains a hindrance to its wider application; notably, hydrogelation requires the incorporation of photoreactive azide groups as cross-linkers and the use of an expensive UV irradiation device that may have harmful effects on normal tissues. Therefore, further modification of chitosan will be required before this material can be used as a suitable agent for submucosal injection in endoscopic techniques.

## 5. Structure of the GI Mucosa and Formation of Polyelectrolyte Complexes

Mucus is a complex, viscous, adherent secretion synthesized by specialized goblet cells in the columnar epithelium that lines all organs that are exposed to the external environment, including the respiratory, GI, reproductive, and oculo-rhino-otolaryngeal tracts [57]. Mucus serves many functions in those locations, including lubrication facilitating the passage of objects, maintenance of a hydrated layer over the epithelium, serving as a barrier to pathogens and noxious substances, and providing a permeable gel layer for the exchange of gases and nutrients with the underlying epithelium [57,58]. Mucus is composed primarily of water (95%), but also contains salts, lipids such as fatty acids, phospholipids, and cholesterol [58], proteins that serve defensive purpose (e.g., lysozyme, immunoglobulins, defensins), growth factors, and trefoil factors. However, the primary component responsible for mucus’s viscous and elastic gel-like properties is the glycoprotein mucin.

Mucins are large, extracellular glycoproteins with molecular weights ranging from 0.5 to 20 MDa. Mucins exist as both membrane-bound and secreted molecules [59], and the two classes share many common features. Both types are highly glycosylated, consisting of ≈80% carbohydrate (primarily *N*-acetylgalactosamine, *N*-acetylglucosamine, fucose, galactose, sialic acid (*N*-acetylneuraminic acid), and mannose) along with traces of sulfate groups. The oligosaccharide chains, which consist of 5–15 monomers, exhibit moderate branching and are attached to the protein core by *O*-glycosidic bonds to the hydroxyl side chains of serine and threonine residues; modifications are arranged in a ‘‘bottle brush’’ configuration around the protein itself [59]. The protein core, which constitutes the remaining 20% of the molecular mass (≈200–500 kDa), is arranged into distinct regions. A central glycosylated region, comprised of multiple tandem repeats rich in serine, threonine, and proline (STP repeats), provides greater than 60% of the amino acid content. Located at the amino and carboxy terminals, and sometimes interspersed between the STP repeats, are regions with an amino acid composition more typical of globular proteins, relatively little *O*-glycosylation, a small number of *N*-glycosylation sites [60], and a high proportion (>10%) of cysteine residues. These cysteine-rich regions contain domains that possess sequence similarity to the von Willebrand factor (vWF) C and D domains, and to C-terminal cystine knot domains [61,62,63]; the Cys-rich domains are involved in dimerization via disulfide bond formation, along with subsequent polymerization of the dimers to form multimers [64]. Overall, mucins are negatively charged at physiological pH, since the polysaccharide side chain ends are composed of fucose and sialic acid residues.

On the other hand, the ECM in digestive organs has three major components: highly viscous mucopolysaccharides (heparan sulfate, keratan sulfate, chondroitin sulfate, mucin, and HA), which cushion cells; insoluble collagen fibers, which provide strength and resilience; and soluble, multi-adhesive ECM proteins (fibronectin, laminin), which bind to mucopolysaccharides in proteoglycans and collagen fibers to receptors on the cell surface [65,66,67]. Different combinations of these components tailor the matrix for different functions depending on the amount of strength (for example tendons), cushioning (for example cartilage), and adhesion required. All ECM components are synthesized intracellularly and secreted via exocytosis. Like mucins, ECM is negatively charged at physiological pH, since the mucopolysaccharide chains harbor both sulfate groups and carboxyl groups [65,66,67].

Basic molecules such as chitosan and protamine are typically complexed (through ionic interactions) with acidic molecules such as mucopolysaccharides, alginate, and fucoidan; the resulting assemblies are termed polyelectrolyte complexes (PECs) [38,68,69,70]. Studies indicate that polyanions and polycations can bind to proteins below and above their isoelectric points, respectively. These interactions can result in nanoparticles, hydrogels, soluble complexes, coacervates, and/or amorphous precipitates. The composition of PECs obtained under various experimental conditions have been investigated for a range of properties, including physical aspects (e.g., the strength and positions of ionic sites, charge density, and rigidity of polymer chains) and chemical aspects (e.g., solubility, pH, temperature, and concentration) [71,72].

Various negatively charged polysaccharides [67,72], proteins [73,74], and lipids [75,76] are present in human and animal tissues. Among these, mucopolysaccharides with anionic sulfate groups and carboxylic groups (e.g., heparin, heparan sulfate, chondroitin sulfate, and HA) are present in various structures and tissues [67,72]. Mucin, which is anionic, is particularly abundant on the gastric mucosa [59]. We hypothesized that positively charged chitosan may be able to form an insoluble hydrogel following electrostatic interactions with negatively charged polysaccharides in the connective tissues, thus precluding the need for azide groups or UV irradiation in generating PCHs [27]. Specifically, we postulated that a chitosan derivative incorporating lactose moieties (linked to the amino groups of glucosamine units, without photoreactive azide groups) should be able to bind with anionic mucopolysaccharides and proteins. The construction and characterization of such a chitosan derivative is expected to contribute to the development of a safe submucosal injectable for use in endoscopic techniques.

## 6. Soluble Chitosan Derivatives for Submucosal Injection

**Figure 6:**
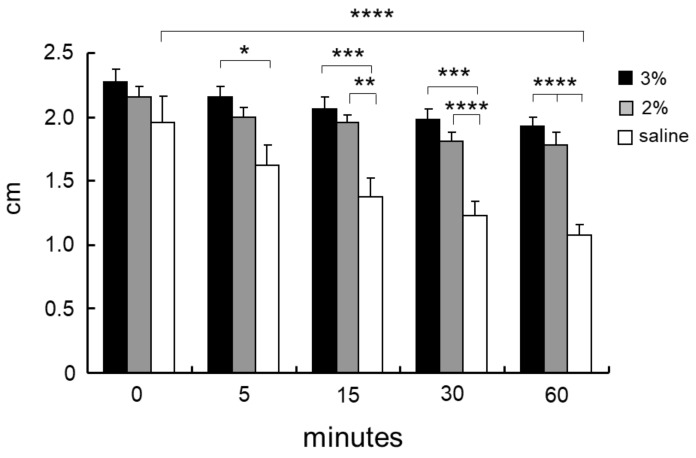
Lifting effect of CH-LA-S. A solution of 3% CH-LA-S (black columns), 2% CH-LA-S (gray columns), or physiological saline (white columns) was injected into the submucosa of *ex vivo* porcine stomach, and the heights of injection volumes were measured at the indicated time point over the subsequent hour. Data are plotted as mean ± SD (n = 5). *p < 0.05, **p < 0.01, ***p < 0.005, and ****p < 0.001 by Tukey post-hoc test. Reprinted from [27], Copyright 2017, with permission from Elsevier.

**Figure 7:**
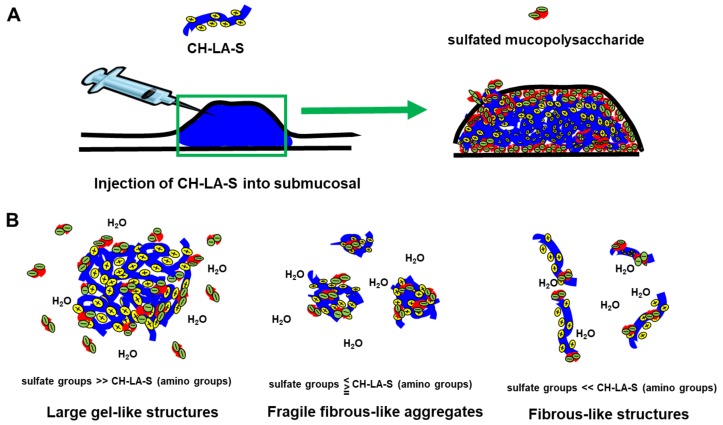
(**A**): Scheme of when CH-LA-S is injected into submucosa. CH-LA-S is adhered to parts in contact with tissues, but the inside of the injected CH-LA-S remains fluid. (**B**): Scheme of different formation patterns due to difference in amount of CH-LA-S and sulfated mucopolysaccharides. Reprinted from [27], Copyright 2017, with permission from Elsevier.

A chitosan derivative (designated CH-LA) was prepared with lactose moieties linked to the amino groups of its glucosamine units [23,24,27]. CH-LA is dissolved in neutral pH solutions such as physiological saline, and 3% and 2% (*w*/*v*) CH-LA solution (CH-LA-S) dissolved in physiological saline were prepared. When 10 mL of 3 and 2% CH-LA-S, and physiological saline were respectively injected into the submucosa of a swine stomach, the heights of tissue volumes immediately after injection were not significant differences. However, the protrusion height of the saline groups decreased after 5 min, and significantly decreased after 60 min. The protrusion heights of 3% CH-LA-S were maintained for more than 60 min, and the mucosal lifting with 3% CH-LA-S were significantly higher than that with only physiological saline (Figure 6) [27]. The injected 3% CH-LA-S adhered to touching parts of tissues in the submucosa, and the inside of the 3% CH-LA-S which was not in contact with the tissues remained fluid (Figure 7A). Moreover, the decreased protrusion heights were primarily due to leakage from the needle hole, and the maintenance of protrusion heights after injection of 3% CH-LA-S due to block the needle hole. In that ex vivo study, it was inferred that CH-LA interacted with negatively charged substrates such as anionic mucopolysaccharides in the muscularis mucosa, submucosa, or muscularis of the gland regions, and with mucin and anionic mucopolysaccharides on the surface mucosa.

CH-LA-S has high water solubility by lactose. The interaction of CH-LA-S with anionic mucopolysaccharides was examined to elucidate the mechanism which CH-LA-S adheres to the tissues in vitro. CH-LA-S is a cationic polymer, and interacts with anionic mucopolysaccharides such as heparin, heparan sulfate, chondroitin sulfate, mucin, and hyaluronic acid. However, the formed structures were differed by the quantity of sulfate groups in polysaccharides [27]. In presence of heparin and chondroitin sulfate having high quantity of sulfate groups, 3% CH-LA-S formed large gel-like structures. In presence of smaller amounts of heparin and chondroitin sulfate, 3% CH-LA-S formed fragile fibrous-like aggregates and fibrous-like structure [27]. The different structures were influenced by not only electrostatic interaction by positive and negative charge but also concentration, pH, and molecular weight [77,78,79]. The complex formation of chitosan with chondroitin sulfate is influenced by water content [80], and the complex formation of chitosan with mucin is also influenced by hydrogen bond and hydrophobic association [81]. However, CH-LA-S do not form a large gel-like structure by the interaction with hyaluronic acid. Hyaluronic acid consisting of d-glucuronic acid and *N*-acetyl-d-glucosamine, is an anionic, nonsulfated mucopolysaccharides. In case of collapses in the balance of amount of positive charges and negative charges, they appear to form fragile fibrous-like aggregates or fibrous-like structures (Figure 7B). The different structures formed by interaction of CH-LA-S with anionic mucopolysaccharides, can be influenced by electric balance of charges by positive and negative charge as well as hydrogen bond and hydrophobic association [27].

Fibronectin is an ECM protein that is present in the stroma around the gastric glands and in the submucosa [74,77], where the molecule functions as a cell adhesion molecule by anchoring cells to collagen and proteoglycan substrates. In addition, fibronectin, a tyrosine sulfated-protein [74,82], may interact with chitosan via similar mechanisms as those observed for the sulfated mucopolysaccharides. Therefore, we speculated that CH-LA-S would also serve as a glue in a matrix that was rich in sulfate groups, given that this chitosan derivative binds to immobilized anionic mucopolysaccharides and sulfated-proteins.

In our preliminary experiment, 3% CH-LA-S was injected into the submucosa beneath a hypothetical tumor in the swine stomach in live, anesthetized pigs. Endoscopic observation revealed sustained lifting of the mucosa, which would allow for successful resection of the hypothetical tumor, additionally the injected CH-LA-S was cross-linked to the submucosal tissue. Thus, following injection of CH-LA-S into tissues with abundant sulfated mucopolysaccharides and proteins such as fibronectin, this chitosan derivative naturally binds to the matrix and forms a gel. However, future in vivo studies will be required to evaluate the safety of CH-LA-S, including its biodegradability, and to compare the effects of CH-LA-S with those of other types of submucosal injections.

## 7. Summary

The endoscopic techniques such as EMR and ESD require proper submucosal injections beneath the tumor to provide a sufficiently high submucosal fluid cushion (SFC) to facilitate clean dissection and resection of the tumor. Proper materials for submucosal injections should provide a SFC sufficient to facilitate clean dissection and resection of tumors. Formation of an SFC has become integral to ESD as well as EMR of large superficial lesions of the GI tract. Although the use of highly viscous solutions such as hypertonic saline with epinephrine, 50% dextrose, and glycerol can help prevent quick dissipations of SFC, these agents tend to cause tissue damage and local inflammation at the site of injection. Other materials such as HA, chondroitin sulfate, and poloxamer 407 can help prevent such complications [43]. Although these last 3 materials provide the longest-lasting cushions among available agents [43,44,45], HA, chondroitin sulfate, and poloxamer 407 are expensive and typically are not available in many endoscopic units. Under such circumstances, we have developed a lactose-modified chitosan (CH-LA) for use as an injectable agent for formation of SFCs in a clinical trial context; the resulting viscous hydrogel soluble at neutral pH without requiring UV irradiation [27]. The use as an injectable agent for generation of a SFC of the chitosan-based material (CH-LA) without UV irradiation exhibits adhesive effects for application as a SFC. CH-LA, the novel chitosan derivative described here, shows improved solubility in neutral solutions, a property that is conferred by the introduction of lactose at the amino groups of chitosan.

We previously studied on viscous Az-CH-LA, which can be converted to a soft, rubber-like hydrogel upon UV irradiation for 30 s [23,24]. A disadvantage of PCH-assisted ESD is the requirement for UV irradiation using an expensive UV fiber for ESD; use of UV may be associated with minor inflammation in residual tissues. Furthermore, the use of UV irradiation creates a risk that PCH-assisted ESD may induce carcinogenesis.
